# Research on the control strategies of data flow transmission paths for MPTCP-based communication networks

**DOI:** 10.7717/peerj-cs.1716

**Published:** 2023-12-06

**Authors:** Zhong Shu, Hua-Bing Du, Xin-Yu Zhu, Shi-Xin Ruan, Xian-Ran Li

**Affiliations:** School of Electronic Information Engineering, Jingchu University of Technology, Jingmen, China

**Keywords:** MultiPath transmission control protocol, MPTCP, Software defined network, InterCluster average classification, Network latency, Network swallowing volume

## Abstract

The performance of multipath transmission control protocol (MPTCP) subflow through the enhancement mechanism of the MPTCP communication is improved. When dealing with multiple MPTCP subflows occupying the same transmission path, critical issues such as selection and optimization of multipath, and efficient scheduling of available multiple tracks are effectively addressed by incorporating the technology called software defined network (SDN) that is constructed based on four key parameters, namely, network transmission bandwidth, transmission paths, path capacity, and network latency. Besides, critical equipment such as the network physical device layer and SDN controller are integrated with the four parameters. So, the network model defines the transmission control process and data information. Considering the predetermined total network bandwidth capacity to select multiple paths, the adequate bandwidth capacity is determined by defining the data transfer rate between MPTCP terminals and MPTCP servers. However, the processing latency of the OpenFlow switch and the SDN controller is excluded. The effective network transmission paths are calculated through two rounds of path selection algorithms. Moreover, according to the demand capacity of the data transmission and the supply capacity of the required occupied network resource, a supply and demand strategy is formulated by considering the bandwidth capacity of the total network and invalid network latency factors. Then, the available network transmission path from the valid network transmission path is calculated. The shortest path calculation problem, which is the calculation and sorting of the shortest path, is transformed into a clustering, Inter-Cluster Average Classification (ICA), problem. The instruction of the OpenFlow communication flow is designed to schedule MPTCP subflows. Thus, various validation objectives, including the network model, effective network latency, effective transmission paths, supply-demand strategies, ineffective transmission paths, shortest feasible paths, and communication rules are addressed by the proposed method whose reliability, stability, and data transmission performance are validated through comparative analysis with other conventional algorithms. Found that the network latency is around 20 s, the network transmission rate is approximately 10 Mbps, the network bandwidth capacity reaches around 25Mbps, the network resource utilization rate is about 75%, and the network swallowing volume is approximately 3 M/s.

## Introduction

A software defined network (SDN) provides a variety of network connection ports for various computing terminals or device terminals (Du et al., 2013; Elaheh, 2023; Linhares et al., 2023), making the research and application of network multipath transmission technology a hot spot (Alcardo & Andrew, 2022; Intidhar, Meriem & Taoufik, 2023; Huang et al., 2023). In SDN networks, multipath transmission technology is inseparable from the multipath transmission control protocol (MPTCP) (Vikas & Samayveer, 2023). This protocol is an extension of the Transmission Control Protocol (TCP) that maximizes the utilization of the link resources of data transmission by enabling multipath data transmission through multiple available paths.

The key technical points of the MPTCP involve bandwidth aggregation of multiple available transmission paths and the implementation of load-balancing strategies across these paths. The primary goal is to enhance the data transmission reliability of the network system. The MPTCP defines the transmission of data through MPTCP subflows; that is, a data transmission task or a transmission file is divided into files and defined as multiple data flows, namely, equivalent to data packets in the TCP communication protocol called MPTCP subflow.

When compared to the SDN, the MPTCP has three drawbacks regarding data transmission path selection and is limited to end-to-end communication, restricting its communication within the physical device layer of the network system to a specific terminal. This limitation prevents real-time monitoring of the operational status of the entire physical device layer. Consequently, multiple subflows of an MPTCP designated for a particular data transmission task may be scheduled onto a single transmission path, resulting in network congestion. This situation exemplifies a failure to utilize the available data transmission paths fully. Furthermore, when a data transmission task is divided into multiple subflows in the MPTCP and assigned to different transmission paths, there may be variations in network latency across these paths. As a result, the received MPTCP subflows at the destination may not maintain their original sequence, leading to a complete disruption of the intended order. This disruption negatively impacts the performance of data transmission.

Finally, the number of the MPTCP subflows divided by a transmission task is fixed, so it is not easy to introduce some autonomous dispatching control strategies from the relationship between data transmission needs and network transmission quotas. Thus, the SDN’s main advantage lies in its unique architecture of the network system, which enables the separation of the network control and data forwarding layers. Moreover, the SDN controller could independently collect information about the structure, and the operational status of the physical device layer and could also gather routing state information from the data forwarding layer. Based on the specific data transmission requirements, the SDN controller can compute an optimal routing path and provide routing control information and forwarding rules to the data forwarding layer to establish the routing and forwarding paths.

Researchers have conducted a series of studies on these three drawbacks of the MPTCP in multipath data transmission. So far, there have been two main research directions on the MPTCP. While the former contains the line of research focusing on controlling the MPTCP subflow transmission paths based on the reliability analysis of network transmission paths, the latter is the research on the application of the SDN. This research direction combines the MPTCP with the SDN to establish a reliable information exchange interface between the MPTCP protocol and the SDN network and aims to achieve unified management and scheduling of routing paths by introducing the SDN.

The first research direction focuses on establishing communication links between network terminals, leveraging the communication interface provided by the MPTCP protocol itself (referred to as the terminal side), which facilitates the implementation of technical improvements. In the second research direction, direct information exchange between the MPTCP protocol and SDN networks is impossible due to their inherent differences. Therefore, the first challenge is to establish a connection interface (the network side) between the MPTCP protocol and SDN networks.

In the reliability analysis of network transmission paths and control of multiple transmission paths, Albert & Fernando (2023) suggested strategies to formulate an MPTCP subflow retransmission. These strategies involve managing and scheduling transmission paths, allocating the optimal route for MPTCP subflows belonging to the same transmission task, and setting up backups for multiple paths to ensure sequential transmission of the MPTCP subflows. Pérez et al. (2023) proposed a methodology based on monitoring the network latency of the MPTCP subflow transmissions to select the transmission path with the shortest latency for the MPTCP subflows. However, this approach was limited to addressing only a subset of shorter MPTCP subflows. Hamza (2022) formulated the guaranteed conditions to cover better transmission quality and reliability, and the autonomous transmission path management and dispatching mechanism were introduced. Amaldi et al. (2016) introduced machine learning models incorporating parameters such as the MPTCP subflow transmission rate, network swallowing volume, and network latency. The goal of training the model on data transmission paths was to select the optimal one. However, it is essential to note that the improved methods above still faced challenges in fully addressing the issues of multiple MPTCP subflows when the same transmission task occupies the same transmission path.

On the other hand, Rost & Schmid (2018) made significant progress in applying the SDN for managing and controlling multipath network transmission and used an SDN network as an experimental platform to test various performance aspects of the MPTCP. So, a solid foundation was provided for subsequent studies on incorporating the SDN into the MPTCP, offering valuable insights and evidence for further advancements in the field. Sherwood et al. (2009) introduced a mechanism in the SDN control layer that enables the MPTCP subflows to autonomously calculate routing paths and control the flow of the MPTCP subflows. This mechanism relies on real-time and rapid monitoring of the operational status of the SDN network, providing the foundation for efficient routing management and control. Drutskoy, Keller & Rexford (2012) also focused on improving the model structure of the SDN by incorporating the MPTCP communication strategies. The key to addressing the problem is to calculate the optimal number of the MPTCP subflows in the transmission path to enhance the network swallowing volume of data transmission paths. This approach aims to achieve routing management and control within the SDN model structure, similar to the method described in Sherwood et al. (2009). However, it is noted that both approaches do not directly address the issues of multiple MPTCP subflows when the same transmission task occupies the same transmission path. Zhu & Ammar (2006) improved the definition of the MPTCP subflows within the SDN framework.

By ensuring that the SDN controller can parse the MPTCP subflows. Then, multiple paths can be utilized for subflow transmission within the same transmission task. Since the selection of numerous transmission paths is an independent process the main objective is to use network bandwidth fully. However, it should be noted that this approach faced challenges in achieving sequential transmission of the MPTCP subflows, which could impact the performance of the network transmission. Yu et al. (2008) utilized the SDN networks to detect the transmission latency of the MPTCP subflows. The criteria of shorter network latency for the MPTCP subflows and more minor differences in completion time among all subflows are considered based on statistical analysis. Hence, routing management, and control rules are established to minimize discrepancies between transmission paths. Therefore, the objective is to enhance the performance of subflow transmission in the transmission paths.

Based on the suggestions derived from the analysis, relying solely on the communication rules of the MPTCP to achieve the multipath transmission of the MPTCP subflows between network terminals poses significant challenges. The complexity of addressing the issues involved in this approach is substantial. The direction of the main breakthrough is to apply the SDN to establish a connection between the SDN networks and the communication rules of the MPTCP. Both research and application of the SDN may have slowed down, but significant research potential in this field still exists.

This article takes the SDN as a premise and focuses on optimizing the multipath transmission of the MPTCP subflows in various adverse network environments. The primary approach is to utilize the SDN control layer to collect information about the data transmission paths and their utilization within the network system, specifically identifying effective transmission paths between communication terminals. Based on factors such as different transmission tasks, network bandwidth resource utilization, and network latency, strategies were formulated to split the MPTCP subflows, and communication interfaces between the SDN control layer and MPTCP were enhanced to enable dynamic routing management and the control of the MPTCP subflows across multiple paths.

The outline of the article is structured as follows: the network model is presented in ‘Network Model’. ‘Key Algorithms’ introduces key algorithms used for dealing with the same types of issues in the literature. Experimental research is conducted and presented in ‘Experimental Results and Discussion’. The research is concluded in ‘Conclusion’.

## Network Model

The SDN network model designed in the article primarily consists of four components: the SDN control layer, the data forwarding layer, the physical device layer, and the MPTCP communication network. The SDN control layer comprises several components, including the SDN controller, the statistics module of the transmission path, the MPTCP subflow control module, and the transmission path control module. The data forwarding layer consists of the SDN network topology, the network operational state module collected by the SDN controller, the OpenFlow communication protocol, and the MPTCP subflow transmission rules. The physical device layer refers to all the managed and utilized OpenFlow switches. The MPTCP network consists of three main components: the terminals and servers of the MPTCP, and the communication protocol.

The statistics module of the transmission path determines the data transmission paths and their capacities based on the network bandwidth and latency requirements of the data transmission. The primary focus is to maximize the network bandwidth and identify all valid data transmission paths. In expanding the data transmission path, all subflows of the MPTCP can only be composed of multiple paths after the encapsulation of a transmission task is abandoned as the same transmission link rule. Then, some paths unrelated to the established and current transmission tasks are incorporated into the practical path.

In preventing of occurring disordered MPTCP subflows, the effective path constraint mechanism is introduced not to occupy the current effective path for the other transmission tasks. Simultaneously, paths with longer latency during data transmission are defined as invalid paths. Hence, enhancing network bandwidth to expand transmission paths would become the primary breakthrough. Therefore, collecting real-time information on network transmission resource utilization is crucial for dynamically updating the determined data transmission paths.

In the MPTCP subflow control module, a strategy for matching between data transmission demand and network resource supply, which is referred to as the supply and demand strategy, is introduced. This strategy is mainly used to determine the number of the MPTCP subflows that need to be transmitted, *i.e.,* to determine the available transmission paths for the MPTCP subflows. Based on the collection of network latency, the longer network latency is defined as invalid latency, and the data transmission paths with longer network latency are defined as invalid paths.

With the supply and demand policy, the invalid paths are deleted in real time when the data transmission process is realized. So, the valid paths and the number of the MPTCP subflows transmitted are updated. Data transmission demand mainly refers to the division rules of the MPTCP subflows and the number of subflows resulting from the division. So, the demand is also used as a condition to determine the capacity of resources such as transmission paths, network broadband, and acceptable network latency. On the other hand, network resource supply mainly refers to the specific capacity of using resources such as effective transmission path, available network broadband, and effective network latency. Collecting the usage status information of real-time network transmission resources is a key prerequisite for real-time updating of the number of MPTCP subflows.

In the control module of the transmission path, the determination of the MPTCP subflow will be arranged for data transmission in each way in the set of valid paths, and the two commands of the available paths and the establishment of a proper network connection will be enabled. Then, the update control policy of the data transmission path is executed, which is essentially called the allocation rule of the MPTCP subflow. The path update of the data transmission is also inseparable from the real-time collection of the status information of resource utilization when the network transmission occurs.

The topology of the whole SDN network model mainly consists of five parts: network transmission broadband (B), network transmission path (P), path capacity (C), network latency (T), network physical layer (R) that consists of all OpenFlow switches, and the SDN controller (S), which is defined as M(B, P, C, T). M(B, P, C, T) mainly provides relevant statistical information for the statistics module of the transmission path, which also includes the information utilization status of the resources when the network transmission occurs. The components are called the network bandwidth, transmission path, and network latency, and three types of information utilization status. The information is transmitted through the OpenFlow communication protocol between the two layers. The SDN controller directly controls the collection module of the network operational state and collects the usage status of the OpenFlow switch in the network’s physical layer. The collection process is targeted at all MPTCP subflows and performs both classification and statistical calculations for all MPTCP subflows within each transmission task, thereby accurately collecting the transmission paths for each transmission task. Statistics are also computed for the transmission paths that are not associated with transmission tasks.

**Figure 1:**
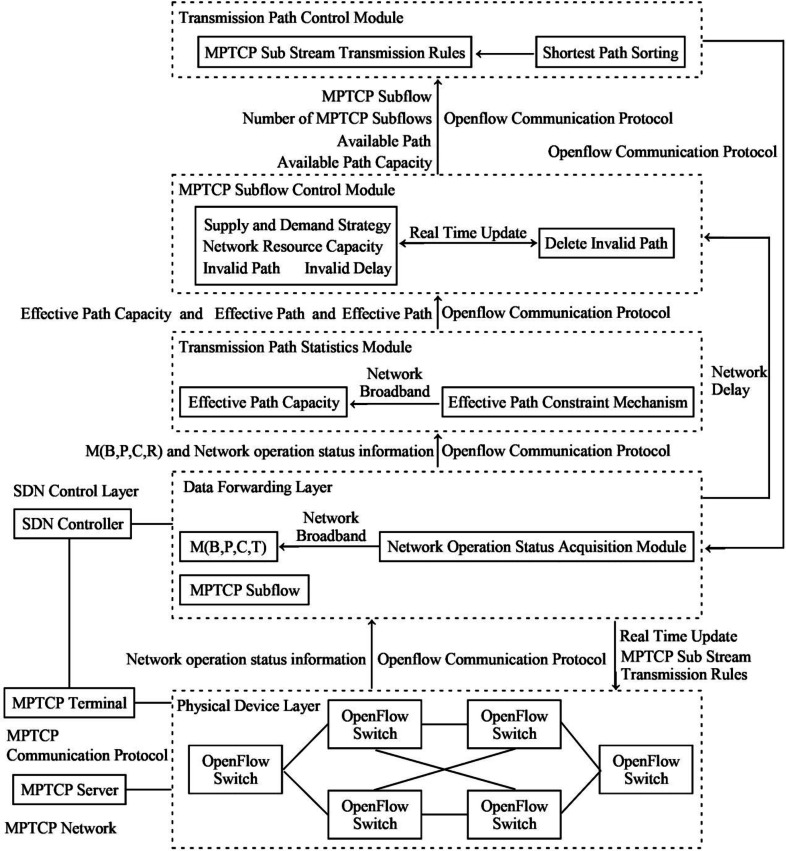
Illustration of the information transmission flow of the network model.

The information transmission flow in the SDN network model is conducted as follows: the SDN controller connects to the physical device layer and constructs the network topology M(B, P, C, T). The resource utilization information of the network transmission is collected through the OpenFlow switch. The M(B, P, C, T) and the resource utilization information of the network transmission are then sent to the transmission path statistics module using the OpenFlow communication protocol. Based on the received resource utilization information from the physical device layer, the available resource information is analyzed and sent to the MPTCP subflow control module. The requirements of the transmission task, and the network latency information from the resource utilization status, the number of MPTCP subflows are determined based on the available resource information. Afterward, the valid MPTCP subflow transmission paths are identified and sent to the transmission path control module. Following the MPTCP substream transmission rules, feedback is provided to the network operational state acquisition module, and the physical device layer executes the MPTCP substream transmission rules. The information transmission process is depicted in Fig. 1.

## Key Algorithms

### Multipath selection algorithm

The proposed multipath selection algorithm primarily entails the calculations of the total bandwidth capacity, network latency, and effective transmission path between the MPTCP terminal and the MPTCP server. Thus, the effective transmission path serves as the final output. The network model is defined as M(B, P, C, T) with the associated parameters detailed in the earlier introduction. The total bandwidth capacity is prioritized based on the maximum bandwidth capacity of the OpenFlow switch at the physical device layer. Suppose that the total bandwidth capacity of the MPTCP terminal is smaller than the maximum bandwidth capacity of the OpenFlow switch. In that case, the maximum bandwidth capacity of the MPTCP terminal is selected as the total bandwidth capacity.

The whole MPTCP network mainly consists of the MPTCP terminals and the MPTCP servers, and the maximum bandwidth capacity of both is the same. The maximum bandwidth capacity of the SDN controller and OpenFlow switch in the SDN network is also the same, so the determination of the total bandwidth capacity only needs to take into account the lowest layer devices of the two types of networks. The OpenFlow switch, the MPTCP terminals, other devices, transmission media, communication protocols, and other factors that realize data transmission in the two types of networks must match the two bottom layer devices in terms of bandwidth capacity. The SDN controller, OpenFlow switch, MPTCP terminal, MPTCP server, the maximum bandwidth capacity of the OpenFlow switch, and the maximum bandwidth capacity of the MPTCP terminal are denoted by *S*, *R*1, *R*2, *R*3, *C*(*R*1), and *C*(*R*2), respectively. The total bandwidth capacity is determined as *C*(*R*) = *C*(*R*1) when *C*(*R*1) ≤ *C*(*R*2), and otherwise, *C*(*R*) = *C*(*R*2). Invalid transmission paths are directly eliminated by using those relations. The type of invalid transmission paths being defined here is the first type, which is not explicitly included or defined in Algorithm 1 (A1) . The invalid paths are only a subset of these invalid paths. The data transmission rate from *R*2 to *R*3, the MPTCP sub-stream capacity of *R*2, the MPTCP sub-stream capacity of *R*3, the effective bandwidth capacity of *R*2 to *R*3 are defined as *V*(*R*2, *R*3), *D*(*R*2), *D*(*R*3), *B*(*R*2, *R*3), respectively within the network latency *T*(*R*2, *R*3). They all are presented in Eq. (1).

(1) \begin{eqnarray*}V(R2,R3)= \frac{D(R3)-D(R2)}{T(R2,R3)} , B(R2,R3)=C(R)-V(R2,R3)\end{eqnarray*}

where *B*(*R*2, *R*3) is defined in terms of all available data transmission paths, *i.e., B*(*R*2, *R*3) is the set of adequate bandwidth capacities, and more detailed implications are explained later.

Regarding the definition of *T*(*R*2, *R*3), the total latency completes the data transmission process between *R*2 and *R*3. Those communication processes are presented as follows: the MPTCP endpoints and the SDN controller, the MPTCP servers and the SDN controller, the internal OpenFlow switch in the physical device layer and the OpenFlow switch, the OpenFlow switch and the data forwarding layer, the data forwarding layer and the transmission path statistic module, the transmission path statistic module and the MPTCP subflow control module, the MPTCP subflow control module and the transmission path control module, the transmission path control module and the data forwarding layer. The communication processes between the data forwarding layer and the physical device layer and the latency of the above communication process can be sequentially defined as *T*(1), *T*(2), *T*(3), *T*(4), *T*(5), *T*(6), *T*(7), *T*(8), and *T*(9). In the network latency statistics, the communications between the MPTCP terminals and the SDN controller and the MPTCP servers and the SDN controller occur directly. These communications’ latency are determined as *T*(1) and *T*(2). Internal OpenFlow switches must be communicated through the SDN controller in the physical device layer. Therefore, the determination of *T*(3) involves two scenarios: the first is based on the latency for collecting operational state information between the SDN controller and OpenFlow switch *T*(*S*, *R*), and the second one employs the latency for feedback of state information between OpenFlow switch and the SDN controller *T*(*R*, *S*). Both *T*(*S*, *R*) and *T*(*R*, *S*) include the transmission of pure state information and the communication for process control that involves the internal operations of the OpenFlow switch and the SDN controller. The processing latencies of the OpenFlow switch and the SDN controller are defined as *T*(*R*) and *T*(*S*), respectively. To precisely define *T*(3), *T*(*R*) and *T*(*S*) included in *T*(*S*, *R*) and *T*(*R*, *S*) are excluded in the article. Thus, the latency of transmitting pure-state information is extracted, which is represented by Eq. (2).

(2) \begin{eqnarray*}T(3)=(T(S,R)-T(S)-T(R))+(T(R,S)-T(R)-T(S))\end{eqnarray*}

where *T*(4), *T*(5), *T*(6), *T*(7), *T*(8), and *T*(9) can be defined precisely in the same way as in Eq. (2). Equation (3) presents:

(3) \begin{eqnarray*}T(R2,R3)=T(1)+T(2)+\cdots +T(9)\end{eqnarray*}

where the data transmission path from *R*2 to *R*3 can be determined based on the value of *B*(*R*2, *R*3).


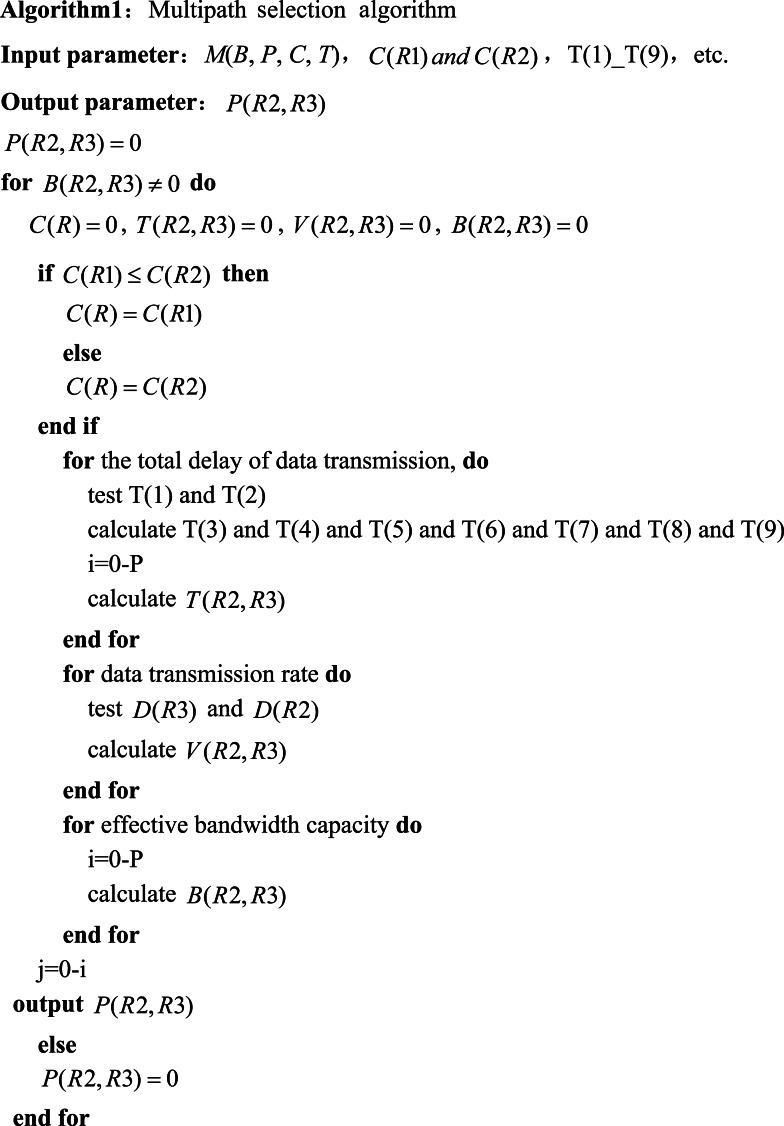


The data transmission path from *R*2 to *R*3 is defined as *P*(*R*2, *R*3), and when *B*(*R*2, *R*3) ≠ 0, there must exist *P*(*R*2, *R*3), otherwise, no such a *P*(*R*2, *R*3) exists. Besides, *P*(*R*2, *R*3) is the set of valid transmission paths called all correct transmission paths.

This study defines the set of all data transmission paths in the network when the algorithm runs for each transmission task *P*. The adequate bandwidth capacity possessed by each effective transmission path is defined as *B*((*i*)(*R*2, *R*3)), where *i* denotes the *i*th path in the set of all data transmission paths *P* from *R*2 to *R*3, and the network latency corresponding to *B*((*i*)(*R*2, *R*3)) is defined as *T*((*i*)(*R*2, *R*3)), where *i* is defined as above, and a certain effective transmission path is defined as *P*(*j*)(*R*2, *R*3), where *j* designates the *j*th path in *i*th. All are expressed in Eq. (4).

(4) \begin{eqnarray*}\begin{array}{@{}l@{}} \displaystyle B(R2,R3)=\sum _{i=0}^{P}B((i)(R2,R3)), T(R2,R3)=\sum _{i=0}^{P}T((i)(R2,R3)),\\ \displaystyle P(R2,R3)=\sum _{j=0}^{i,i\in P}P((j)(R2,R3)) \end{array}\end{eqnarray*}

The number of path entries *P*(*R*2, *R*3) is first used to select from 0 to all the path entries, then select *j* from 0 to *i*. *P*(*R*2, *R*3) realizes the update in conjunction with *j*.

The main code of the multipath selection algorithm based on the above expression is presented as follows:

### An available MPTCP path optimization algorithm

The optimization algorithm for the number of MPTCP subflows is designed in this study. The number of the MPTCP subflows is used to determine all the available transmission paths that can transmit the MPTCP subflows among the set of valid transmission paths output *P*(*R*2, *R*3) from A1 . The available transmission paths are the optimal number of MPTCP subflows to be determined. The computation is mainly based on formulating the supply and demand strategy that computes the data transmission demand capacity of a task and the supply capacity of the network resource. The demand capacity of the data transmission refers to the transfer rate *V*(*R*2, *R*3) of the MPTCP subflow, while the supply capacity of the network resource refers to *B*(*R*2, *R*3). The data transmission process fully utilizes the existing network resources. However, this scenario is rarely achievable but is used as the ideal condition in practical applications. Nevertheless, it remains the pursuit goal of the proposed algorithm in the manuscript. *V*(*R*2, *R*3) > *B*(*R*2, *R*3) indicates insufficient network resources, so network congestion may be expected. However, it does not necessarily mean that MPTCP subflow transmission is entirely impossible. This is a situation that the proposed algorithm aims to avoid as much as possible. On the other hand, *V*(*R*2, *R*3) < *B*(*R*2, *R*3) indicates that the data transmission process does not fully utilize the available network resources. This is a situation that the proposed algorithm aims to control. Therefore, the requirement is determined based on the experimental results and considering a 60% or higher achievement rate of network resource utilization. The proposed algorithm defines the conditions that meet the strategy requirements of the supply–demand as follows:

(5) \begin{eqnarray*}V(R2,R3)\leq B(R2,R3) {and}\nolimits  V(R2,R3)/B(R2,R3)\geq 60\%\end{eqnarray*}

Defining the invalid network latency as $\stackrel{\leftarrow }{T}(R2,R3)$, and combining with the previous precise definition of the effective network latency *T*(*R*2, *R*3), it can be determined that the first condition belonging to the invalid network latency is denoted by $\stackrel{\leftarrow }{T}(R2,R3)\gt T(R2,R3)$. To achieve the goal of available path optimization, it is also necessary to introduce the parameter of the network latency difference as the second condition for determining the invalid network latency. The network latency difference and the average network latency are defined as *T*(*ϕ*) and $\bar {T}(R2,R3)$, respectively. $\bar {T}(R2,R3)$ is determined by experimentally detecting the network latency of multiple MPTCP subflows of a transmission task. The number of divided MPTCP subflows is denoted by *n*, then, the average network latency is expressed as $\bar {T}(R2,R3)={\mathop{\sum }\nolimits }_{n=0}^{P}T((n)(R2,R3))/n$, which gives the value of $T(\phi )=T(R2,R3)-\bar {T}(R2,R3)$. Thus, the maximum value of *T*(*ϕ*) is taken as the invalid network latency. The algorithm’s determination condition of the ineffective network latency is expressed in Eq. (6).

(6) \begin{eqnarray*}\stackrel{\leftarrow }{T}(R2,R3)\gt T(R2,R3) {and}\nolimits  \max \nolimits T(\phi )\end{eqnarray*}

The available transmission paths for MPTCP subflow transmission are defined as $\vec{P}(R2,R3)$. The algorithm first determines what $\vec{P}(R2,R3)$ contains based on Eqs. (5) and (6) by following the determination of the number of bars *k* in $\vec{P}(R2,R3)$. They all are presented in Eq. (7).

(7) \begin{eqnarray*}\begin{array}{@{}l@{}} \displaystyle B(R2,R3)=\sum _{j=0}^{i,i\in P}B((j)(R2,R3)), T(R2,R3)=\sum _{j=0}^{i,i\in P}T((j)(R2,R3)),\\ \displaystyle \vec{P}(R2,R3)=\sum _{k=0}^{j,j\in i,i\in P}\vec{P}((k)(R2,R3)) \end{array}\end{eqnarray*}

*B*(*R*2, *R*3) and *T*(*R*2, *R*3) are updated for the corresponding parameters in Eq. (4), and $\vec{P}(R2,R3)$ are also realized for *P*(*R*2, *R*3), and usable transmission paths, *k*, are selected among the number of *j* valid transmission paths.

The main code of the available MPTCP path optimization algorithm based on the above definition is presented as follows:

### Multipath management scheduling algorithm

The multipath management scheduling algorithm suggested in the manuscript is primarily used to allocate MPTCP subflows that need to be transmitted to available transmission paths for multipath data transmission, focusing on developing MPTCP subflow allocation rules and defining control instructions for *R*2 to *R*3. The MPTCP subflow allocation rules are based on the first principle of the shortest path within the available transmission paths, considering both the capacity and quantity of network resources. The control instructions for the transmission paths utilize the OpenFlow communication protocol.


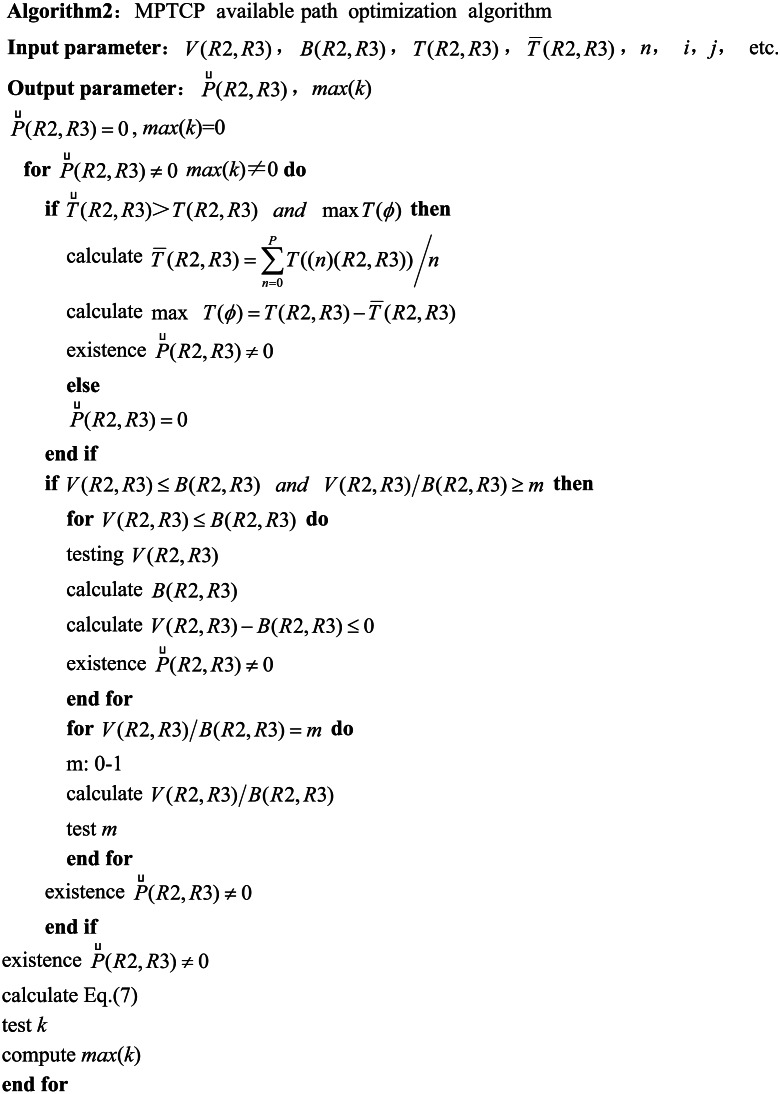


The shortest path selection first needs to resolve the classification problem of the capacity and quantity of the network resources. For the classification, the fastest path set is defined in descending order from the largest to the smallest, and the MPTCP subflow transmission is prioritized for the large capacity and quantity of the network resources. In this article, the supply capacity of the network resource, effective network latency, and available transmission paths are all included in the scope of network resources, and the network resources of different natures are comprehensively categorized and sorted. The clustering algorithm is the best one to organize the parameters of different natures, which has two clusters: intracluster and intercluster (Chowdhury, Rahman & Boutaba, 2012; Cheng et al., 2011). Due to the different types of class parameters, the classification based on the intercluster approach is chosen.

The parameters of the shortest path mainly involve the main parameters in Eq. (7). *B*(*R*2, *R*3) denotes the capacity of the network resource supply, *T*(*R*2, *R*3) represents the effective network latency, and $\vec{P}(R2,R3)$ characterizes the available transmission paths, which can be specified as the capacity parameters, namely, *i*, *j*, and *k* are the parameters showing the number of transmission paths specified as the three quantities (Heller, Sherwood & McKeown, 2012; Demirci & Ammar, 2014; Gong et al., 2016; Haghani, Bakhshi & Capone, 2018).

The definition of the shortest path refers to the maximum value among the six parameters mentioned above. The sorting process of the shortest path involves arranging the six parameters from the maximum to the minimum. As the three capacity parameters indicate, the meaning of their nature is entirely different and they do not belong to the same type of parameter. The three quantity parameters, though belonging to the same kind of parameter, do not belong to the same category as the three capacity parameters (Li et al., 2018; Martini, Gharbaoui & Castoldi, 2023). So, the proposed algorithm necessarily combines the above six parameters to calculate the shortest path. If the above six parameters are calculated independently from the maximum to the minimum and sorted by the average value, the calculation process includes many steps. Therefore, the Inter-Cluster Average Classification (IAC) algorithm described in the article can be used to perform the classification for the shortest path in a single step. Then, the results can be sorted in descending order. Thus, the set of shortest paths, $(G \left\vert (B,T,\vec{P},i,j,k) \right. )$, is defined as follows:

(8) \begin{eqnarray*}\begin{array}{@{}l@{}} \displaystyle (G \left\vert (B,T,\vec{P},i,j,k) \right. )= \frac{1}{ \left\vert B \right\vert \cdot \left\vert T \right\vert \cdot \left\vert \vec{P} \right\vert } \\ \displaystyle (\sum _{j=0}^{i,i\in P}B((j)(R2,R3)),\sum _{j=0}^{i,i\in P}T((j)(R2,R3)),\sum _{k=0}^{j,j\in i,i\in P}\vec{P}((k)(R2,R3)))\\ \displaystyle \max \nolimits (G \left\vert (B,T,\vec{P},i,j,k) \right. )\rightarrow \min \nolimits (G \left\vert (B,T,\vec{P},i,j,k) \right. ) \end{array}\end{eqnarray*}

In Eq. (8), $\max (G \left\vert (B,T,\vec{P},i,j,k) \right. )\rightarrow \min (G \left\vert (B,T,\vec{P},i,j,k) \right. )$ is the sorting result of the shortest path, and the result is controlled by the parameters *i*, *j*, and $k. \left\vert B \right\vert $ and $ \left\vert T \right\vert $ and $ \left\vert \vec{P} \right\vert $ denotes the membrane of the three vector parameters of $({\mathop{\sum }\nolimits }_{j=0}^{i,i\in P}B((j)(R2,R3)))$ and ${\mathop{\sum }\nolimits }_{j=0}^{i,i\in P}T((j)(R2,R3))$ and ${\mathop{\sum }\nolimits }_{k=0}^{j,j\in i,i\in P}\vec{P}((k)(R2,R3))$, respectively. It is possible to assign available paths to each MPTCP subflow based on the sorted result in Eq. (8).

The execution flow of OpenFlow communication commands is as follows: OpenFlow switch receives the first MPTCP subflow (in the first MPTCP subflow) → OpenFlow switch feeds back to the data transmission request of the SDN controller → SDN controller analyzes the transmission demand → SDN controller calculates the shortest available path based on the demand → SDN controller ranks the shortest available paths from the largest to the smallest → SDN controller allocates the transmission path for the first MPTCP subflow in the order of the shortest available path → SDN controller ranks the shortest available paths from largest to smallest → SDN controller assigns the first MPTCP subflow to the first ranked transmission path → OpenFlow switch forwards the MPTCP subflow to other OpenFlow switches in the following available paths → OpenFlow switch receives the second MPTCP subflow and feeds it back to the SDN controller → SDN controller analyzes this MPTCP → The SDN controller analyzes the MPTCP subflow and determines the relationship between the MPTCP subflow and the previous subflow (whether it is the same transport task, the sequence numbers are consecutive, and it is the sequence number of the last subflow) → the SDN controller assigns the second-ordered transport path for the MPTCP subflow → the transport of all MPTCP subflows is completed in accordance with the transport method of the second MPTCP subflow → the SDN controller completes the transport of all MPTCP subflows in the second MPTCP subflow based on the sequence number of the last MPTCP subflow in the first MPTCP subflow.

The SDN controller disconnects the communication connection after completing the allocation of available paths for the last MPTCP sub-stream and determining that the transmission of the sub-stream is completed. Note that the above communication process does not include the process between the MPTCP terminal and the MPTCP server.

**Figure 2:**
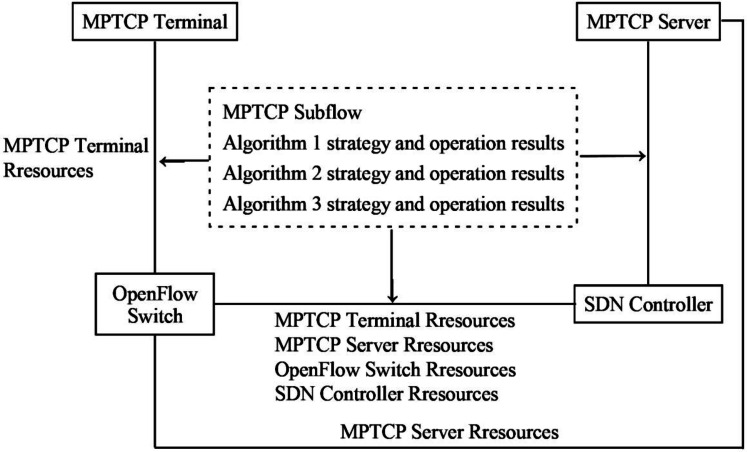
Diagrammatic representation of the data transmission process of the network system.

The main code for the proposed multipath management scheduling algorithm is outlined based on the above definitions as follows:

The data transmission process of the above three algorithms is expressed as follows: MPTCP terminal sends the data transmission task to the OpenFlow switch, OpenFlow switch receives the data transmission task, requests the SDN controller for task feedback, and submits the basic situation and utilization information of network resources concurrently. The SDN controller completes the counting of effective paths for the MPTCP sub-flow based on A1 and completes the allocation of multipath for the MPTCP subflow based on Algorithm 3 (A3) . The SDN controller uses A1 to compute the statistics of multiple valid paths of the MPTCP subflow, calculates multiple available routes of the MPTCP subflow according to Algorithm 2 (A2) , and completes the multipath allocation of the MPTCP subflow according to A3 . The SDN controller submits the data transmission task of the MPTCP terminal by sending the data to the MPTCP server and the MPTCP subflow transmission rules and finally realizes the data transmission process between the MPTCP terminal and the MPTCP server. Figure 2 shows the data transmission process of the whole network system.


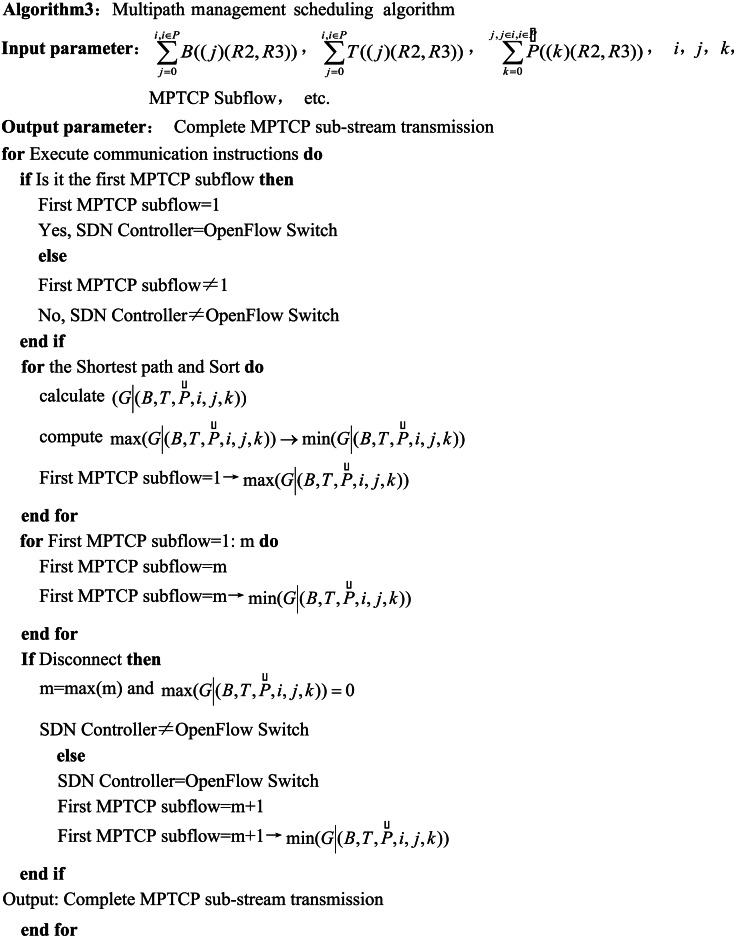


## Experimental Results and Discussion

### Experimental settings and parameters

**Figure 3:**
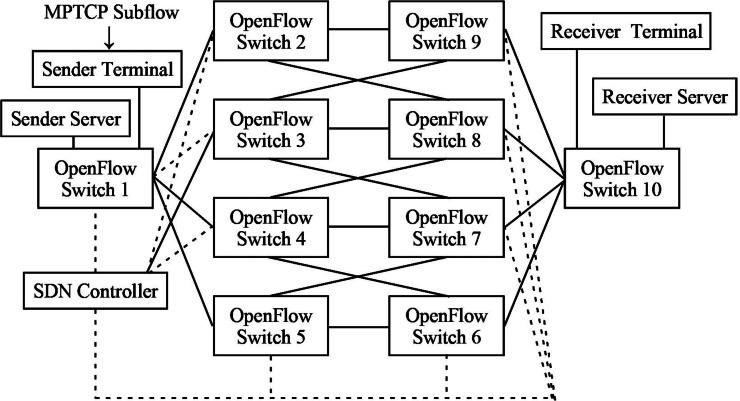
Diagrammatic representation of the experimental network topology.

The experimental network topology is shown in Fig. 3. The implemented algorithms include A1 , A2 , and A3 , and the proposed algorithm, called Algorithm 4 (A4). A1–A4 is mainly used for the self-evaluation of the experiment. The algorithms involved in the comparison experiments are the other four algorithms proposed in Amaldi et al. (2016), Drutskoy, Keller & Rexford (2012), Zhu & Ammar (2006), Yu et al. (2008), Chowdhury & Boutaba (2010), Yoo et al. (2023), Alkmim, Batista & da Fonseca (2013) and Feamster, Gao & Rexford (2007), which are referred to as A5, A6, A7, and A8, respectively. Also, A5–A8 is mainly used for the comparative evaluation with A4.

 The indicators for evaluating the performance of the network and the proposed algorithm mainly include network swallowing volume, data transmission rate, network bandwidth, network latency, and network resource utilization. The simulation equipment consists of two MPTCP terminals (terminals used and sending and receiving), two MPTCP servers (servers used for sending and receiving), 10 OpenFlow switches, and one SDN controller. The SDN controller and a video file of about 2G are selected as the transmission file. The main experimental content, experimental protocol, and evaluation parameters are defined below:

In the topology definition of the SDN network model, it is only necessary to include two parameters, the network broadband and transmission path, which can be defined as M(B, P). The network broadband and transmission path are denoted by *C*(*R*) and *P*(*R*2, *R*3), respectively. The topology of the whole SDN network model for the experiment includes the network broadband *C*(*R*), transmission paths (total number of paths equals p bars) based on Fig. 3 are calculated, which is p =(8)!/(8-4)! =1,680. Path capacity *P*(*R*2, *R*3), network latency *T*(*R*2, *R*3), and the four other parameters are characterized by M(B, P, C, T). In this case, the four primary devices in the network’s physical layer, MPTCP terminals, MPTCP servers, OpenFlow switches, and SDN controllers are directly integrated into the four parameters.

Experiment 1 (E1) compares the network transmission performance by constructing the M(B, P) and the M(B, P, C, T) network models, and the main parameters for evaluating the network transmission performance are called the data transmission rate (V, Mbps), the network bandwidth (H, Mbps), the network latency (T, s), the network resource utilization (O, %), and the network swallowing volume (Q, M/s).

V represents the variation of the data transmission rate, which is the difference between the received data transmission rate denoted by V2 = *V*(*R*2, *R*3) and the sent data transmission rate denoted by V1 = *B*(*R*2, *R*3), then V =V2-V1 is expressed. H represents the network bandwidth variation used to differentiate from the network bandwidth B, which is the difference between the applied bandwidth of the experimental detection H2 and the total bandwidth H1 = *C*(*R*), denoted by H =H2-H1. T denotes the actual detection time of the experiment for completing the transmission of all MPTCP sub-streams. O is determined based on the network broadband’s usage capacity of the experimental detection, H2, the path usage capacity of the observed detection, P2 = $\vec{P}(R2,R3)$, the total bandwidth, H1, and the total capacity of the transmission path, P1 = *P*(*R*2, *R*3), and O =(H2+P2)/(H1+P1).

Q is obtained based on the values of V, H, and T and is related to the values of V1 and H1, expressed by Q =((V1+V)+(H1+H))/T. Subsequent experiments for setting the main parameters are uniformly denoted by using V (V1 and V2), H (H1 and H2), P (P1 and P2), O, and Q. However, the calculated statistics will differ depending on the meanings of the experimental parameters and methodologies that are subsequently used. The values of network swallowing volume defined in this article are not as significant as the ones extracted from the findings of conventional network swallowing volume in Fig. 3.

In A1 , two experiments, E2 and E3, are carried out. Based on the previous definition of data transmission delays T(S, R) and T(R, S) of the OpenFlow switch and the SDN controller, the proposed algorithm excludes the two parameters of the two devices’ processing delays TI and T(S), which are used to compute the data transmission rate V(S-R). In general, the data transmission rates defined by T(R) and T(S) are the factors, and their data transmission rate is defined as V(S, R). E2 compares and analyzes the performance of A1 for the mentioned two cases above. The evaluation performances of the main network transmission parameters are called data transmission rates V(S-R) and V(S, R). It also includes the four evaluation parameters of H, P, O, and Q. According to the conventional effective path selection method, the effective paths are selected by directly selecting i out of all data transmission paths p as the effective paths based on the calculation process of both the bandwidth capacity and network latency. According to the rules of A1 , Ii paths are selected from the first p based on the definitions of the effective bandwidth capacity and the corresponding network latency. Then, *j* effective paths are selected Irom *i* paths. E3 is used to compare and analyze the performance of A1 regarding the above two scenarios. The evaluation performance of the main network parameters are called T, V, H, O, and Q, respectively.

Then, the next two experiments, E4 and E5, are carried out. Based on the previous definition of meeting the requirements of the supply and demand policy and the utilization of network resources whose score is 60% or more, the size of the capacity is determined between V1 and H2 using one of the three cases of <, =, and >, the determination of O is carried out by evaluating the parameters of T, V, H, and Q in E4. Hence, the invalid paths are defined twice in A1 and A2 . The first definition is based on the value of the total bandwidth H1, and the paths just <H1 are directly defined as invalid transmission paths.

The second definition is based on the computation of the invalid network latency $\stackrel{\leftarrow }{T}(R2,R3)$. The network latency difference *T*(*ϕ*) and the paths with a large difference in network latency are defined as invalid transmission paths too. In E5, the two cases of defining invalid transmission paths for the first and second time are compared to verify the network transmission performance of the two algorithms. So, the main parameters for evaluating the network transmission performance are called T, V, H, O, and Q, respectively.

In A3 , two experiments, E6 and E7, are conducted. Based on the six parameters, which are called three capacity parameters and three quantity parameters, are involved in the definition of the shortest path. The commonly used method is employed to determine the shortest path by taking the average between the maximum and minimum scores of each parameter and then calculating the standard deviation of the six parameters. Most practices have not selected all six parameters to make computations yet. However, it is usually taken from the network model. When the supply capacity of the network resource and transmission path capacity are defined, A3 selects six parameters. On the other hand, the proposed algorithm obtained the shortest path by using Eq. (8).

E6 evaluated data transmission performance based on the two fastest path calculation methods above. Based on the definition of MPTCP sub-streams under the SDN network platform and the communication transmission rules of the SDN network, A3 improved the two rules, and E7 defined the conventional method as B3. The improved process, A3 , is used to compare the network transmission performance using the two methods. Thus, the performance evaluation of the main network transmission parameters concerning both E6 and E7 are T, V, H, O, and Q, respectively.

Moreover, to evaluate the performance of the proposed method, A4, it is necessary to compare it with the four other algorithms, namely A5, A6, A7, and A8 (Amaldi et al., 2016; Drutskoy, Keller & Rexford, 2012; Zhu & Ammar, 2006; Yu et al., 2008). The performance evaluation of the primary network transmission parameters is called again T, V, H, O, and Q, respectively.

The selection of the evaluation parameters of the experiment and methods mentioned above demonstrated a high degree of integration with the proposed algorithm. Each experiment employs a comprehensive range of quantitative (algorithmic-specific) and qualitative (performance-based evaluation) parameters to the best extent possible.

### Experimental results and discussion

The experimental statistics are shown in Tables 1 through 8 based on the above experimental program.

**Table 1:** Experimental data for E1 statistics.

Model	Parameter					
	V2 (Mbps)	V1 (Mbps)	H2 (Mbps)	H1 (Mbps)	P2 (Mbps)	P1 (Mbps)
M(B, P)	6	10	12	20	8	16
M(B, P, C, T)	8	10	14	20	12	16

Model	Parameter				
	T (s)	V (Mbps)	H (Mbps)	O (%)	Q (M/s)
M(B, P)	35	−4	−8	56	0.51
M(B, P, C, T)	32	−2	−6	72	0.68

In the E1 experiment concerning V1, H1, and P1 parameters, their values are necessarily the same if the same type and number of devices are used regardless of the way to construct the results of the network model, which is the basic premise of this experiment. The network model M(B, P) only includes the capacity of the SDN network bandwidth and the number of transmission paths. The network bandwidth capacity consists of the path quantity, which represents the main components of the entire network concisely and comprehensively. This representation is a standard approach for describing the network structures of the topology in the current context. The constructed network model, M(B, P, C, T), includes the parameter C, which describes the capacity of P and helps calculate the overall transmission capacity of all hardware devices in the entire network, as it encompasses the devices from both MPTCP and SDN networks. Table 2 shows that T is linked to the full range of experimental parameters. The parameter Q is defined as a comprehensive parameter of the network’s transmission performance, allowing for an almost thorough evaluation of the entire network’s performance.

Among numerous research achievements, T is also considered a core parameter and must be included in the network model. When evaluating the performance of the network transmission using solely the separate actual values of V2, H2, and P2 without considering the inherent relationship between the original values V1, H1, and P1 the assessment of the actual operational state of the entire network is affected. However, this impact may not be significant. By introducing the three differential parameters V, H, and P, their values could be applied to both O and Q, which enriches the importance of both O and Q. Thus, this comprehensive approach enhances the credibility of the evaluation performance of the network transmission. The values of V =V2-V1 and H =H2-H1, theoretically, could only take 0s as the most desirable maximum value. Therefore, the values of experimental and practical applications are necessarily less than 0 when negative values exist. The smaller the performance value of the network transmission is, the worse the result would be. The calculation of the Q-value is the same as the intrinsic values of V1, H1, and P1, respectively to establish a link. The obtained statistics in Table 2 show that the M(B, P, C, T) model is superior to the M(B, P) one in terms of data transmission rate, network bandwidth, network latency, network resource utilization, and network swallowing volume.

**Table 2:** Experimental data for E2 statistics.

Model	Parameter							
	V(S, R) (Mbps)	V(S-R) (Mbps)	V (Mbps)	H (Mbps)	P (Mbps)	O (%)	Q (M/s)	T (s)
T(R2, R3)	6	/	−3	−6	8	76	58	29
T(R2, R3)-T(S)-T(R)	/	8	−2	−5	12	89	76	25

In the E2 experiment, the results of O and Q are equal to above 80% and 60%, respectively, and are used to evaluate and validate the results of T. The experiments were conducted to compare the two cases of whether the OpenFlow switch is included in the SDN controller network’s processing latency. Due to the different storage capacities and processing capabilities of the two devices, their impact on the network latency is so significant that the algorithms could not be fairly evaluated. While T(R2, R3) represents the latency that includes the processing delay of the two types of devices, T(R2, R3)-T(S)-T(R) represents the latency without considering the processing delay of the two types of devices. The experimental data indicates that the results of both O and Q are close to 80% and 60%, respectively when considering the factors related to the two types of devices. However, A1 shows ideal results for O, Q, and T when not considering the factors associated with the two types of devices.

**Table 3:** Experimental data of E3 statistics.

Route	Parameter							
	V (Mbps)	H (Mbps)	O (%)	Q (M/s)	T (s)	P (Mbps)	i (strip)	j (strip)
(p)-(i)	−4	−7	71	55	39	6	1543	/
(p)-(i)-(j)	−3	−5	83	70	31	10	1543	957

In the E3 experiment, which focuses on evaluating the effective number of data transmission paths (strips) through the final values of both O and Q. In Table 4, (p)-(i) indicates that i paths are selected from the total number of transmission paths p for data transmission, and (p)-(i)-(j) indicates that i paths are the first selected from the total number of transmission paths p. Then, j paths are selected from i paths for data transmission. Theoretically, the more occupied data transmission paths are, the larger the capacity provided by the number of transmission paths would be, the higher the occupancy of network resources, and the more significant the amount of data swallowing would be. A1 relies on the definition of the number of effective paths as a prerequisite to consider the two important factors per the adequate path capacity and the effective network latency. Moreover, (p)-(i) is based on the premise of using all paths without truly considering the effectiveness of the paths.

**Table 4:** Experimental data for E4 statistics.

Capacity	Parameter					
	V2 (Mbps)	V1 (Mbps)	H2 (Mbps)	H1 (Mbps)	P2 (Mbps)	P1 (Mbps)
V1>H2	6	13	11	20	10	18
V1=H2	7	13	13	20	14	18
V1<H2	9	13	16	20	15	18

Capacity	Parameter				
	T (s)	V (Mbps)	H (Mbps)	O (%)	Q (M/s)
V1>H2	27	−7	−9	55	0.63
V1=H2	19	−6	−7	71	1.05
V1<H2	15	−4	−4	82	1.67

In the E4 experiments, based on the relationship between the data transfer rate V1 and the network resource capacity H2, the values of both O and Q are evaluated, and the primary basis for the evaluation is assigned to O ≥ 60%. The statistics in Table 5 show that when V1 > H2, O < 60%, but close to 60%, *Q* = 0 and 63 Mbp/s. Concluded that A2 has the same performance as the sound data transmission in the case of insufficient resource capacity. When V1 ≤ H2, there is a significant improvement in data transfer performance.

**Table 5:** Experimental data for E5 statistics.

Method	Parameter					
	V2 (Mbps)	V1 (Mbps)	H2 (Mbps)	H1 (Mbps)	P2 (Mbps)	P1 (Mbps)
<H1	8	13	13	20	12	18
T(Φ)	10	13	18	20	14	18
<H1+T(Φ)	10	13	16	20	13	18

Method	Parameter				
	T (s)	V (Mbps)	H (Mbps)	O (%)	Q (M/s)
<H1	21	−5	−7	71	1.00
T(Φ)	15	−3	−2	84	1.87
<H1+T(Φ)	17	−3	−4	76	1.53

In the E5 experiments, three methods of determining invalid paths using A1 , A2 , and A1+A2 are compared to verify the network transmission performance of A2 . The primary evaluation is based on the values of T, O, and Q, respectively. In Table 6, <H1 indicates that A1 is used to remove the invalid path directly. T(Φ) indicates that A2 is used to determine the invalid path by defining the invalid network latency, and <H1+T(Φ) indicates that a combination of A1 and A2 is used to describe the invalid path. The statistics in Table 6 show that the values of T, O, and Q are more desirable in the <H1 condition. The T(Φ) condition is the best network transmission effect, and the design goal of A2 can be achieved in the <H1+T(Φ) condition. The main reason for the superiority of T(Φ) over <H1+T(Φ) is that the network latency is small, and the complexity of the operation is low, but the invalid paths are selected without being filtered by <H1, which may incorporate a small number of invalid paths into them. Hence, it may include a small number of invalid path selections. Therefore, <H1+T(Φ) is the best method to select invalid paths.

**Table 6:** Experimental data for E6 statistics.

Method	Parameter					
	V2 (Mbps)	V1 (Mbps)	H2 (Mbps)	H1 (Mbps)	P2 (Mbps)	P1 (Mbps)
Average	9	15	18	25	13	20
Formula8	11	15	21	25	17	20

Method	Parameter				
	T (s)	V (Mbps)	H (Mbps)	O (%)	Q (M/s)
Average	28	−6	−7	69	0.96
Formula8	21	−4	−4	84	1.52

The E6 experiment is used to compare the two methods of calculating the shortest path in A3 and verify the performance of the network transmission of A3 . The basis of the primary evaluation uses T, O, and Q values. Table 6 shows the direct average of the six parameters, namely, network resource supply capacity, effective network latency, available transmission path capacity, *i*, *j*, and *k*. Equation (8) is used to calculate the averages of the six parameters in the intercluster. Also, the experimental statistics show that the average network time is longer, more computation steps are needed, and the data transmission performance is not favorable. Based on the evaluation method of the previous experiments, A3 has good data transfer performance, and the values of both O and Q are improved by employing both A1 and A2 .

**Table 7:** Experimental data for E7 statistics.

Algorithm	Parameter					
	V2 (Mbps)	V1 (Mbps)	H2 (Mbps)	H1 (Mbps)	P2 (Mbps)	P1 (Mbps)
B3	6	15	12	25	9	20
A3	12	15	20	25	16	20

Algorithm	Parameter				
	T (s)	V (Mbps)	H (Mbps)	O (%)	Q (M/s)
B3	41	−9	−13	47	0.44
A3	23	−3	−5	80	1.39

The E7 experiment is used to evaluate the performance of the network transmission using two algorithms, B3 and A3 , which are mainly based on the values of T, V, H, O, and Q. The algorithm B3 refers to the use of international MPTCP standard and OpenFlow communication protocols for data transmission, and A3 indicates the use of improved communication rules, which are generally compatible with international standard protocols. The experimental statistics show that the network latency value of A3 is significantly low. Hence, the network resources are effectively utilized, and the network swallowing volume is improved dramatically.

**Table 8:** Experimental data for E8 statistics.

Algorithm	Parameter				
	T(whole) (s)	V(whole) (Mbps)	H(whole) (Mbps)	O(whole) (%)	Q(whole) (M/s)
A5 (Amaldi et al., 2016)	23	11	21	78	3.12
A6 (Drutskoy, Keller & Rexford, 2012)	33	8	18	61	1.27
A7 (Zhu & Ammar, 2006)	38	6	16	43	0.81
A8 (Yu et al., 2008)	26	8	20	67	1.83
A4	21	10	24	78	3.28

In the E8 experiment, the advantages of the proposed algorithm are validated by comparing it with the algorithms mentioned in the relevant references. Since there are fundamental differences between the proposed algorithm A4 and A5 (Amaldi et al., 2016), A6 (Drutskoy, Keller & Rexford, 2012), A7 (Zhu & Ammar, 2006), and A8 (Yu et al., 2008), respectively, the definitions of the whole network latency T, network transmission rate V, network bandwidth H, network resource utilization O, and network swallowing volume Q in this experiment are completely different from the definitions used in the previous experiments. These parameters specifically focus on the entire process of data transmission completion. The primary experimental environment remains the same as defined earlier.

In the article, A4 refers to the sequential execution of A1 through A3 . The experimentally obtained data shows that the introduction of machine learning models for training and determining key parameters plays a crucial role in improving the performance of the network transmission in A5 In A6, controlling the number of MPTCP subflows helps maintain stable swallowing volume of the network. A7 expands the data transmission paths and emphasizes the sequential transmission and reception of MPTCP subflows as key factors that affect the performance of the network transmission. A8 focuses on network latency, implementing multipath transmission routing control, and effectively improving performance. When compared to the four algorithms mentioned above, the proposed algorithm, A4, has significant advantages in various performance metrics. However, a slightly lower network transmission rate than those in A5 was observed.

The proposed algorithm achieves a desirable data transmission performance mainly due to the following reasons. (1) A simplified network model: The algorithm consolidates multiple functional modules, incorporates all key parameters into the network topology structure, and primarily focuses on implementing data transmission by using the OpenFlow communication protocol while ensuring compatibility with the MPTCP. (2) Comprehensive selection of multiple paths: The algorithm breaks away from conventional expansion ranges, selects more effective data transmission paths, considers a wide range of parameters related to path selection, and enables more efficient path selection. (3) Consideration of data transmission requirements and network resource supply: The algorithm compares and selects available paths based on data transmission demands and network resource availability, accurately identifies and captures essential control parameters, and ensures the selection of feasible data transmission paths. (4) Enhanced MPTCP subflow division and OpenFlow communication rules: The algorithm improves the division of the MPTCP subflows and optimizes OpenFlow communication rules. Applying clustering and averaging operations achieves the selection, prioritization, and scheduling of the shortest transmission paths, thus effectively controlling the orderly transmission and reception of MPTCP subflows.

## Conclusion

The optimization of the transmission path of the MPTCP subflow is carried out using the SDN technology to build a network model. A multipath data transmission algorithm, including effective path selection, efficient path optimization, and available path control, is proposed. The primary findings are summarized as follows:

(1) In the constructed network model, just the primary network management functions of a general SDN controller are used. However, the modules such as MPTCP subflow control, transmission path statistics, and transmission path control are independently designed in the article. Thus, the separation between the SDN controller and the functional modules is achieved. Higher compatibility with MPTCP networks is also achieved, which is better than when the function modules + SDN controllers are fused. As the communication protocols of the MPTCP network and the SDN network are very different, integrating them directly through a simple interface transfer will inevitably corrupt data transmission. Through the data forwarding layer inherent in the SDN network the connection of the three modules in sequence is realized. The unified OpenFlow communication protocol can be independently used to transmit information. In terms of the connection between the MPTCP and the SDN networks, the practice of connecting the MPTCP terminal and the MPTCP server to a particular independent functional module is abandoned, and the MPTCP terminal and the MPTCP server are directly related to the SDN controller. The constructed network model focuses only on the three control objectives of network broadband, transmission path, and network latency in the article. Instead of comprehensively incorporating the SDN network management and control objectives, the subsequent algorithms’ operational complexity is significantly reduced.

(2) The multipath selection algorithm designed in this study incorporates only four parameters in the entire network model, namely, network transmission bandwidth, network transmission paths, path capacity, and network latency. These parameters describe the network’s physical layer, including all OpenFlow switches and corresponding SDN controllers through the network transmission bandwidth and latency. This optimization dramatically improves the topology structure of the network. In the proposed algorithm, the first category of invalid paths is defined by directly detecting the maximum bandwidth values of the MPTCP terminals, MPTCP servers, and OpenFlow switches. Under the premise of a specific transmission task, the entire set of available paths in the network is computed and analyzed based on the data transmission rate, the implementation of the effective path constraint mechanism involves determining the effective transmission paths based on the available bandwidth capacity and network latency of all transmission paths. Hence, two rounds of calculations are needed. The total latency of the data transmission process is precisely calculated, and a straightforward and concise computation method is used to determine the parameters of the entire data transmission process, thus compensating for the complexity introduced by the calculation of total latency.

(3) The proposed MPTCP available path optimization algorithm mainly employs four parameters: the MPTCP subflow transmission rate, the network resource supply capacity, the ineffective network latency, and the effective transmission paths. The restrictions are formulated based on supply and demand strategy, and effective network latency. Afterward, the available transmission paths are selected from the effective transmission paths. The need for the available transmission paths is designed in the supply and demand strategy by comparing and assessing the data transmission capacity, the demand, and the supply capacity of occupied network resources while keeping the utilization rate in mind. Effective network latency is achieved by formulating the constraints of ineffective network latency, which reduces the number of control parameters and the burden of the computational process. Additionally, while considering the limitations of ineffective network latency in the multipath selection algorithm, the parameters of the average network latency are introduced to calculate the difference in the network latency, further enhancing the reliability of the selection of the available transmission paths. By defining or calculating the network resource capacity at the global level and the precise quantity of network resources locally, the method also decreases its operational complexity.

(4) The proposed multipath management scheduling algorithm utilizes clustering methods to calculate the parameters of different network resources and employs the average ICA algorithm to classify and rank the shortest paths. Incorporating the clustering approach, it accurately computes the available shortest transmission path, enabling the optimal ranking of available transmission paths. Additionally, allocation strategies are devised for available transmission paths based on specific transmission tasks. Improving the partitioning rules of the MPTCP subflows and optimizing communication between the SDN controller and OpenFlow switches establishes the communication connection upon completing the first MPTCP subflow transmission. However, it is interrupted after the completion of the last MPTCP subflow transmission, eliminating the need for a separate design of communication connection and interruption commands. The subflow partitioning rules for the transmission task are defined within the first MPTCP subflow, determining the total number of subflows and assigning sequence numbers. The SDN controller interrupts the communication connection based on the sequence number of the last subflow. The algorithm introduces additional computational parameters in the calculation engine of the shortest path. It adds fields in the definition of the MPTCP subflow to describe the relationship between subflows, increasing the computation’s complexity. However, by reducing the steps in the OpenFlow communication process, the balance of the entire computation process is maintained.

(5) An experimental plan is established to evaluate the performance of the suggested algorithm internally and compare it to other algorithms by incorporating the required quantitative and qualitative factors. The experimental findings show that the proposed method is highly reliable and stable.

Directions for subsequent research could be (1) Probabilistic computing methods can be used to determine more effective data transmission paths; (2) Deep learning models can be integrated into the computation and scheduling of effective data transmission paths and available transmission paths.

## Supplemental Information

10.7717/peerj-cs.1716/supp-1 Supplemental Information 1 Code

## Additional Information and Declarations

### Data Availability

The following information was supplied regarding data availability: The code is available in the Supplemental File.
